# Will farmers intend to cultivate Provitamin A genetically modified (GM) cassava in Nigeria? Evidence from a *k*-means segmentation analysis of beliefs and attitudes

**DOI:** 10.1371/journal.pone.0179427

**Published:** 2017-07-11

**Authors:** Adewale Oparinde, Tahirou Abdoulaye, Djana Babatima Mignouna, Adebayo Simeon Bamire

**Affiliations:** 1 Research Fellow, International Food Policy Research Institute, Washington, DC, United States of America; 2 International Institute of Tropical Agriculture, Ibadan, Nigeria; 3 Obafemi Awolowo University, Ile Ife, Nigeria; University of Vermont, UNITED STATES

## Abstract

Analysis of market segments within a population remains critical to agricultural systems and policy processes for targeting new innovations. Patterns in attitudes and intentions toward cultivating Provitamin A GM cassava are examined through the use of a combination of behavioural theory and *k-*means cluster analysis method, investigating the interrelationship among various behavioural antecedents. Using a state-level sample of smallholder cassava farmers in Nigeria, this paper identifies three distinct classes of attitude and intention denoted as low opposition, medium opposition and high opposition farmers. It was estimated that only 25% of the surveyed population of farmers was highly opposed to cultivating Provitamin A GM cassava.

## Introduction

Genetically modified (GM) technology has a tremendous potential to transform agricultural productivity and food security in developing countries [1–3]. [4] have shown that GM technology such as Bt Cowpea has the potential to increase expected net social welfare in Africa. It has even been predicted that GM crops will increase yield, be more readily available and cheaper by 2050 [5]. This technology is increasingly gaining policy attention across the continent [6]. Yet there is a divided world between anti-GM activists and Pro-GM lobbyists about the potential of the technology to improve welfare of the rural poor in the 21^st^ century [7,8].

Micronutrient deficiency, also known as hidden hunger, is one of several developmental challenges facing many developing countries. One in three people in the world suffer from hidden hunger, caused by lack of minerals and vitamins in their diets, which leads to negative health consequences [9]. Biofortification, the process of breeding and delivering staple crops with higher micronutrient content provides a comparatively cost-effective means for increasing the daily adequacy of micronutrient intake and for combating micronutrient deficiency in many developing countries[10, 11]. Biofortification can be achieved either through conventional breeding or genetic modification. While only conventionally bred biofortified Provitamin A cassava varieties have been officially released and disseminated in Nigeria, some Provitamin A GM cassava varieties are currently under trials in confined fields through the BioCassavaPlus (BC+) project (not HarvestPlus). This paper therefore attempts to understand farmers’ immediate behavioural reactions to the concept of GM and Provitamin A.

Several studies have investigated public opinions about GM and demand for GM crops across countries but most of these have been on consumers [12–15]. Research on farmers’ attitude towards GM crops has not been on a rapid pace compared with that on consumers. This trend is also similar in Africa, where most studies have shown mixed opinions among consumers [16, 17]. However, information on the opinion of rural farmers who are the potential producers of GM crops can be instrumental in shaping more evidence-based frontier in the debate on the importance of GM crops for Africa. Even when the technology becomes politically acceptable, adoption among smallholders will determine its success in potentially improving food security since the continent is dominated by smallholder farmers [18] also noted that *ex ante* diagnostic research is important for creating enabling environment for GM products in smallholder agriculture.

Few studies such as [19] linked influence of attitude (based on perceived risk and benefit) to the actual adoption of Bt Maize among smallholder farmers in South Africa where policy on GM technology has been operational. In countries where policy on GM is under consideration, the relationship between farmers’ attitude and potential adoption of GM crops can only be viewed through predictions based on *a priori* theory. [20] treated farmers as producers and consumers simultaneously in exploratory models applied to investigate attitudes toward GM banana in Uganda. While this study considers willingness to buy as the ‘consumer component’ and opinion statements on agronomic traits, risks, benefits and institutional trust as the ‘producer component’, these are indirectly linked to the potential adoption of GM. Although these studies assume that attitude and behaviour are strongly related, the two variables are not directly linked. A number of antecedents influence behaviour and as such, indirectly linking perceptions about risk and trust to behaviour omits the holistic view of processes important for making predictions concerning behavioural reactions to the introduction of GM crops [21]. Therefore, a direct and objective measure of the potential adoption in terms of willingness to commit resources to the cultivation of GM crops is important to expatiate on how perceive benefit and risk factors influence farmers’ intentions toward the technology in Africa.

In addition to the exploration of the link between attitude (based on perceived risks and benefits) and actual or potential adoption of GM crops, the current literature is characteristised by a number of studies across countries examining segments within a target population. Evidence from these studies have suggested various profiles of farmers’ opinions concerning GM, including the supporters, skeptics, environmentally and socially influenced, government trusted, food safety concerned and strong opponents [6, 20, 22, 23]. Building upon this literature, this paper operationalises a theoretical framework that captures various antecedents that link to behavior, that is, the Theory of Planned Behaviour (TPB) [24] to: (i) examine the influence of attitude (constructed based on perceived risk and benefits) on rural smallholder farmers’ intentions and (ii) identify patterns in their opinions by using the case of Provitamin A GM cassava in Nigeria (see Appendix - comment 1). GM is still in a policy consideration stage in Nigeria and GM crops are only cultivated in confined fields.

In order to explore patterns in farmers’ opinions about GM, a *k-*means cluster analysis was conducted where factors affecting farmers’ position regarding GM cassava cultivation are also examined under a multinomial logit framework. Attitudes toward benefits and risks associated and how they are related to farmers’ intention to adopt Provitamin A GM cassava are assessed. The belief system considered in constructing attitude ramifies into six categories: (i) ethical concerns about nature and religion; (ii) agronomic trait regarding pest resistance; (iii) environmental risk and input usage; (iv) food safety; (v) nutritional benefit; and (vi) profitability and affordability.

These areas are considered important based on evidence emerging from the literature assessing individuals’ attitudes toward GM. Studies have shown that pest resistance and input requirement are important agronomic traits that influence sensitivity of demand for GM crops [25]. [26] also predicted demand for GM oil seed based on farmers’ trade-offs among economic and agronomic traits, the result of which shows that profitability significantly affects GM adoption. [15] showed that the vitamin A content has a large utility effect on consumer demand for Provitamin vitamin A GM cassava in Brazil. We also expect the nutritional trait to positively influence farmers’ attitudes toward cultivating Provitamin A GM cassava since micronutrient deficiency is still a major public health problem in Nigeria, where about 30 percent of children under five are vitamin A deficient [27]. A more recent study by [28] found that vitamin A deficiency was prevalent among 17% of children under five in the southern state of Akwa-Ibom. Ethical concerns regarding the relevance of GM technology within the space of religion and culture are considered important since Nigeria is traditionally a religious society and this may have an important bearing on farmers’ perception of GM and GM cassava. Nigeria has over 270 ethnic groups practicing a tri-religion system composing of Christianity, Islam and traditional religions. Although Islam is majorly practiced in the northwestern and northeastern geopolitical zones while Christianity is majorly practiced in the south, there is no clear scientific representation and numerical distribution of these religions among the Nigerian population [29]. There is a general perception, however, that about half of Nigerians are Muslims, about 40–45% are Christians while 5–10% practice traditional religions [30].

The next section begins with a description of Provitamin A GM cassava and the role of BC+ in its development. The third section discusses the theoretical framework used and the application of *k-*means cluster analysis technique in segmenting farmers’ opinions about GM cassava. Results are then discussed on the relationship between TPB variables and factors affecting farmers’ positions regarding the cultivation of Provitamin A GM cassava. Conclusions are drawn regarding the implication of farmers’ attitudes and intentions for the adoption of Provitamin A GM cassava in the future.

### Provitamin A GM Cassava

Cassava is the second most important food staple in Africa after maize, and it is consumed by more than 200 million people in Africa south of the Sahara, who derive more than 50 percent of their calories from the crop [31]. Conventional breeding has led to the development of Provitamin A cassava varieties with high beta-carotenoids and cassava mosaic disease resistance. While conventionally bred Provitamin A cassava varieties have economically important drivers for adoption such as high yield, early maturity and disease resistance, insufficient genetic variation in cassava may limit the ability of this approach to reach full target biofortification objectives [32]. As a result of this, the BC+ has the overall goal of developing Provitamin A GM cassava using genetic modification approach. BC+ aims at developing cassava varieties that can provide the minimum daily requirement for Provitamin A in a 500g meal for an adult and 250g meal for a child [33]. Some Provitamin A GM cassava varieties are currently under trials in confined fields in Nigeria by experts at the National Roots Crops Research Institute, Umudike. Phase II of the BC+ project is still seeking regulatory approval. Once approved, the project will progress to on-farm trials of GM technology. Studies such as this one are therefore important to inform the potential adoption of Provitamin A GM cassava in Nigeria. This could assist policymakers and donors in taking informed decisions while assessing the potential impact of GM as well as in developing effective risk communication and marketing strategies.

## Methodology

### Theoretical framework

In understanding GM product-based behaviour among stakeholders, random utility and consumer choice theories have been fostered where it is usually assumed that individuals’ attitudes and intentions are based on attributes of the good; and that individuals will behave rationally by having a positive attitude towards options that provide maximum utility [20, 34]. Mixed results obtained from various consumer and producer studies across countries suggest that the behavioural and opinion formation process goes beyond product attributes. Studies applying the theory of reasoned action (TRA) have shown that knowledge and information about the product determine attitude [35].

Generally, informational asymmetry among the public regarding GM is an important component of the behavioural process [36]. Several experimental auction studies have found that people’s discount for GM products differ depending on their perception of negative or positive information. [37] found that consumers with negative information have up to 38% discount for GM products while those who received positive information have about 4% discount. This finding was confirmed in another study by [14] where consumers with negative information have about 29% discount for golden rice and those with positive information have 16% discount. Positive information about the environmental, social and health benefits may minimize consumer risk concerns and vice versa [38]. Therefore, the framing of product information and *a priori* knowledge about the product have important roles in the process taken by stakeholders to form their opinions regarding GM technology [39]. As a result, a general practice of presenting opinion statements on both benefit and risk of GM is common in the literature investigating public perceptions and attitudes toward GM crops [20, 23].

Most studies examining producer-and-consumer’s awareness, knowledge and attitude toward GM—conducted in African countries with and without GM experience [16, 19, 40, 41, 42]; have shown that public awareness about GM could be high in some cases but knowledge about different aspects of GM is generally low. There are high information costs and poor knowledge about GM technology in many African countries. Therefore, the provision of information about the technology is warranted as a precedence for farmers to form opinions about their adoption intentions for Provitamin A GM cassava. However, apart from information, perception of social pressure (subjective norm) is one of the key antecedents of intention formation which can also shape stakeholder opinions about GM crops [21]. As such, the TPB can be utilised to explain different aspects of behavioural antecedents.

#### Theory of planned behaviour (TPB)

The TPB is one of the most widely applied behavioural theories in explaining intention and its relationship with behavior [24]. It hypothesizes that an intention is jointly determined by attitude (*A*), perception of control over a decision or an action (perceived behavioural control—*PBC*) and perception of social pressure from significant referents whose opinions are important to an individual (subjective norm—*SN*). Applications of the TPB have shown that an intention predicts an actual behaviour but this theory has not been widely applied to the understanding of the potential adoption of GM technology in sub-Saharan Africa, which is not surprising due to the novel nature of GM crops [21, 43]. However, the predictive power of the TPB has been established in several social and health contexts [44, 45, 46]. Several meta-analyses have reported a large effect size for the relationship between intention and actual behaviour [47–49].

The TPB is an extension of [50]’s Theory of Reasoned Action (TRA), which proposes that attitude determines intention which in turn is determined by subjective norms (*SN*) and perceived behavioural control (*PBC*). *Attitude (A)* refers to a person’s overall evaluation of a behaviour i.e. positive or negative sentiment towards performing a behaviour and it occurs as a consequence of an individual’s belief about various features of the outcome (behavioural beliefs, *b*_*i*_). Therefore, attitude is determined by behavioural beliefs weighted by the outcome evaluation, *e*_*i*_ of how desirable the consequences are (*A* = ∑(*b*_*i*_ x *e*_*i*_)). *SN* is the overall perceived social pressure based on the perception of those individuals whose opinions are important to an intention or a behaviour [51]. *SN* is a measure of an individual’s beliefs about how significant referents would expect someone to behave (normative beliefs, *n*_*i*_) weighted by the person’s motivation to comply (*m*_*i*_) (*SN* = ∑(*n*_*i*_ x *m*_*i*_)). *PBC* is the extent to which a person feels to have control to enact a behaviour [52]. This refers to how difficult a farmer thinks it is to enact the behaviour of adopting Provitamin A GM cassava in the future or actualise an intention to adopt it. Therefore, *PBC* is determined by how much personal control cassava farmers have to actualise their adoption intentions (control beliefs–*c*_*i*_) weighted by how confident they feel about being able to actualise this control (influence of control beliefs—*p*_*i*_) (*PBC* = ∑(*c*_*i*_ x *p*_*i*_)). As proposed by the TPB, the level of variance in farmers’ intentions to adopt Provitamin A GM cassava can be explained by various antecedents of behaviour discussed above.

In the existing literature investigating patterns in public opinions about GM crops, there is a general practice of applying factor analysis technique to reduce a long list of opinion statements on attitude into some combinations that are utilised to perform cluster analysis [20, 23]. As discussed above, the TPB has demonstrated that opinion pattern will be different depending not only on attitude but also on *SN* and *PBC*. Therefore, instead of preceding cluster analysis with a variable reduction technique like factor analysis, we apply the TPB where all the behavioural variables are already distinctly identified.

### k-means cluster analysis

A *k*-means cluster analysis technique is adopted in identifying different clusters of cassava farmers with similar characteristics in terms of their opinions toward cultivating Provitamin A GM cassava (attitude, SN and PBC). This technique is usually considered as appropriate for a small sample size [53]. The *k-*means clustering method groups a number of cases, *i* into *K* distinct clusters based on measures of inter-cluster dissimilarity and intra-cluster similarity without any assumption about the underlying distribution of the observations. The cluster dissimilarity is measured in terms of the distances of individual cases to the cluster centre [54]. Sample vectors’ objects are classified into distinct clusters, *K < n*, where *n* is the number of observations. If *m*_*j*_ is the mean of variable vectors in cluster *j*, then the distance between an observation and cluster mean (*m*_*j*_) is measured as

$$d=\sum_{j=1}^{k}\sum_{i=1}^{m}\left|\right|x_{i}^{\left(j\right)}-m{\left|\right|}^{2}$$ (1)

and minimised to yield non-overlapping *K* clusters. The squared Euclidean distance is the chosen metric to measure the distance (*d*) between two data objects (*p*, *q*) for a vector of variables (*x*′). The optimal number of clusters is determined by calculating the Calinski-Harabasz pseudo *F-*statistic (CH) as the cluster validity index. CH is the ratio of the mean square for *K* clusters divided by the mean square of the residuals [55] and can be formally represented as

$$CH=\frac{\left[\frac{R^{2}}{\left(K-1\right)}\right]}{\left[\frac{\left(1-R^{2}\right)}{\left(n-K\right)}\right]}$$ (2)

where *R*^2^ is the difference between inter-cluster variance or total sum of squared distances to the overall centroid (*SST*) and intra-cluster variance (*SSE*) or sum of squared distances of observations to the cluster’s centroid divided by inter-cluster variance i.e.

$$R^{2}=\frac{\left(SST-SSE\right)}{SST}.$$ (3)

### Consent and ethical statement

The study was a part of larger varietal adoption study, which was approved by the ethics committee of the Obafemi Awolowo University in Nigeria. The research used a non-invasive method. Farmers were informed about the study and were asked for their consent to participate. They verbally provided their consents to participate.

### Sampling and data collection

**Fig 1:**
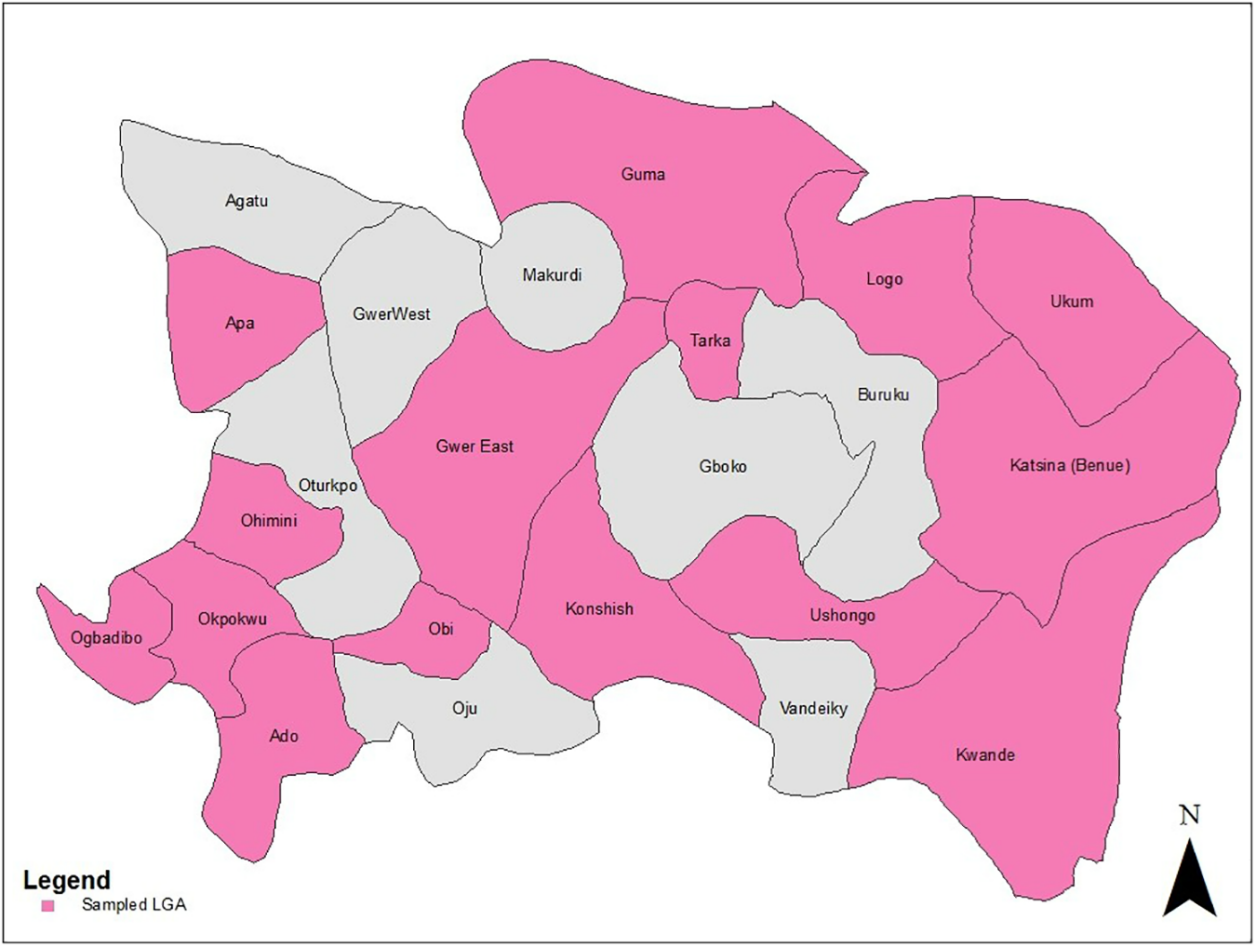
a: LGAs where the selected EAs are located in Benue State. b: LGAs where the selected EAs are located in Oyo State.

This study was conducted between February and May 2012 in Benue (north-central region) and Oyo states (south-western region) which are among the top ten cassava producing states in Nigeria in terms of area under cassava cultivation. Farmers were selected using a two-stage cluster sampling design, with enumeration areas (EAs) (smallest National Bureau of Statistic sampling units) being the clusters. EAs are the first-stage sampling units while households are the second-stage sampling units. From each state, 18 EAs were randomly selected proportionate to size. Fig 1 shows the local government areas (LGAs) in which the selected EAs are located in each state. Total number of EAs in each state and number of households in each EA were obtained from the National Bureau of Statistic (NBS) master sampling frame and the 2008 joint World Bank-NBS household list respectively. From each EA, eight households were then randomly selected. From each household, the person responsible for cassava production decision making was selected, resulting in a sample size of 144 farmers per state.

In conducting the study, we preceded the opinion survey with information transfer since the literature has shown that farmers’ knowledge about GM is limited especially in rural communities (Section 1). Therefore, information on what GM and Provitamin A GM cassava are, was first communicated to each consented farmer as an approach to allow farmers construct their opinions. Information about GM and Provitamin A GM cassava contained in Box 1 (see S1 Box) was developed to be neutral in order to avoid influencing opinion formation among farmers. This is why information about rich and alternative sources of vitamin A in diet was also communicated to farmers.

Trained sociologists utilised artistic props in communicating information about Provitamin A GM cassava to each farmer. As an opinion formation process, each farmer was left alone for about an hour after the information was passed across so that the respondent can have some time to reflect on information received. We recognised that farmers’ low level of knowledge about GM would raise concern about the validity of their opinions after receiving a brief information within a short period of time. However, we consider this enquiry to be valid for two reasons. First, we are interested in farmers’ immediate behavioural reactions to the concept of GM and Provitamin A GM cassava. Second, we consider an exploration of these immediate reactions as a first step towards shedding lights onto farmers’ opinions about the potential adoption of Provitamin A GM cassava in Nigeria. Once farmers gain more information over time, further research would be warranted in understanding the dynamics, which is beyond the scope of the current paper.

Subsequently, a structured questionnaire was administered face-to-face with each of the farmers. The questionnaire utilised for the survey consisted of the first section on a series of questions on household socio-economics while the second section composed of a series of questions investigating TPB variables. Questions utilised in this regard were developed by following [24] and [56]’s manual for constructing a questionnaire based on the TPB. Opinion statements utilised for measuring the TPB variables are shown in Table 1.

**Table 1:** Opinion statements on Farmers’ attitude towards Provitamin A GM cassava.

s/n	Belief Category	Behavioural belief statement (*b*_*i*_)	Likert scale for *b*_*i*_	Outcome evaluation statement (*e*_*i*_)	Likert scale for *e*_*i*_
***i*.**	*Ethical concerns*				
	Nature	Developing GM cassava goes against nature	Strongly disagree (1) to strongly agree (5)	Cultivating GM cassava would make me feel as though I am doing something unnatural	Very unlikely (-2) to very likely (2)
	Religion	Developing GM cassava goes against my religion	Strongly disagree (1) to strongly agree (5)	Cultivating GM cassava would make me feel as though I am acting against my religious beliefs	Very unlikely (-2)to very likely (2)
***ii***	*Agronomic trait regarding pest resistance*				
	Pest Resistance	Cultivating GM cassava will increase pest resistance	Strongly disagree (1) to strongly agree (5)	The potential increase in pest resistance resulting from cultivation of GM cassava will be…	Very undesirable (-2) to very desirable (2)
***iii***	*Environmental risk and input usage*				
	Pesticide usage	Cultivating GM cassava will increase the need for pesticides and pesticide usage	Strongly disagree (1) to strongly agree (5)	The potential increase in pesticide usage resulting from cultivation of GM cassava will be…	Very undesirable (-2) to very desirable (2)
	Fertiliser Usage	Cultivating GM cassava will reduce the need for fertiliser usage	Strongly disagree (1) to strongly agree (5)	The potential reduction in the need for fertiliser use resulting from cultivation of GM cassava will be…	Very undesirable (-2) to very desirable (2)
***iv***	*Food safety*				
	Consumer safety	Consuming GM cassava will be safe for human beings	Strongly disagree (1) to strongly agree (5)	Consuming GM cassava would make me feel as though I am eating something unsafe for my health	Strongly disagree (-2) to strongly agree (2)
***v***	*Nutritional benefit*				
	*Nutrition*	Consuming GM cassava will be good for me and my family because it will be more nutritious	Strongly disagree (1) to strongly agree (5)	The potential improvement in nutrition resulting from consumption of GM cassava will be…	Very undesirable (-2) to very desirable (2)
***vi***	*Profitability/affordability*				
	Price	GM cassava will be cheaper than conventional varieties	Strongly disagree (1) to strongly agree (5)	The potential affordability of GM cassava compared to conventional cassava varieties will make GM cassava	Very undesirable (-2) to very desirable (2)

Note: Total number of question items = 8; *b*_*i*_′s likert scale range = 1 to 5; *e*_*i*_′s likert scale range = -2 to +2; Maximum Attitude Score for 8 items = (*Max*_*bi*_ * *Max*_*ei*_) * 8 = *b*_*i*_*e*_*i*_ * 8 = (5*±2)*8 = ±80.

Institutional trust is an important factor underlying attitudes toward GM [12, 15]. A multi-study review of trust measurement has shown that the concept is broad and complex while it lacks a unified definition [57]. It is possible to view trust from both cognitive perspective of a pre-conditioned basis for social relation [58] and [59]’s behaviouralist concept of ‘rational trust’. Under a social relation perspective, trust has been treated as endogenously conditioned on reliability, reciprocity, fairness and ethical behaviour of an agent [60]. Each of these aspects of trust requires a detailed attention which is difficult to operationalise in a short survey as in the current paper. Rather than treating trust as a catch-all concept, we included a question specifically measuring farmers’ level of trust in government agricultural extension agents as a proxy for institutional trust.

## Results and discussions

### Socioeconomic characteristics of respondents

Socio-economic characteristics of the respondents are presented in Table 2. More respondents claimed to have heard about GM (12%) in Oyo state than in Benue (2%) state. Similarly, 10% claimed to have heard about GM cassava in Oyo while less than 2% have heard about GM cassava in Benue. In both states, 66% of respondents are male with an average age of about 48 years. On average, respondents in Benue have significantly larger household size, more years of education and higher number of under five-children compared with respondents in Oyo.

**Table 2:** Socioeconomic characteristics by state.

Variable	Oyo	Benue
% Respondent is male	66.4	66.0
% Respondent planted cassava (last 12 months before survey)	94.5	97.9
% Respondent heard about GM before survey	11.6	2.1
% Respondent heard about GM cassava before survey	9.6	1.4
% Respondent is household head	65.1	69.4
% Married monogamy	58.2	50.0
% Married polygamy	32.2	33.6
% Single never married	4.1	5.7
% Widowed	5.5	9.3
% Separated	0.0	0.7
% Divorced	0.0	0.7
	**Mean (Std. dev.)**	**Mean (Std. dev.)**
Respondent’s Age (years)	50.9 (15.0)	45.3 (15.0)
Respondent’s education (years)	4.2 (4.9)	9.1 (5.0)
Household size	7.4 (4.1)	9.9 (5.1)
Number of children under five years of age	1.1 (1.3)	3.0 (2.7)
Area of land cultivated with cassava (ha)	1.9 (2.3)	3.0 (8.2)

### Pattern of farmers’ intentions to cultivate provitamin A GM cassava

Measures of TPB variables reported here are considered as farmers’ immediate behavioural reactions after receiving information about GM, and should be treated as such. The intention of cassava farmers toward cultivating Provitamin A GM cassava was measured directly by a question examining the percentage of their cassava land area that they will be willing to dedicate to the cultivation of Provitamin A GM cassava if and when made available (i.e. the percentage that a respondent is willing to cultivate with GM out of her/his currently cultivated cassava land area). On average, farmers surveyed in Benue state cultivated about 3 hectares of land with cassava while farmers surveyed in Oyo cultivated 2 hectares of cassava land area (Table 2).

Table 3 shows pattern of intentions among cassava farmers in each state based on their immediate reactions to the concept of GM. About one third of farmers in both states were willing to cultivate Provitamin A GM cassava with 41–50% of their currently cultivated cassava land area. In Benue state, almost 40% of farmers were willing to dedicate up to half of the cultivated cassava land area to Provitamin A GM cassava. However, in Oyo state about 22% were willing to dedicate as high as 100% of their cassava land area to Provitamin A GM cassava cultivation while only about 7% were willing to do the same in Benue state. This regional disparity in intention suggests the reason as to why on average, farmers in Oyo state were willing to cultivate Provitamin A GM cassava with about 53% of cassava land area while Benue farmers on average were willing to dedicate about 44%.

**Table 3:** Share of cassava land area farmers were intending to dedicate to the cultivation of Provitamin A GM cassava.

**Share of cassava land area (% (in range) of currently cultivated cassava land area that respondents were willing to allocate to GM cassava)**	**% Farmers**		
	Benue (N = 144)	Oyo(N = 144)	Both states(N = 288)
0–5	9.84	0.00	4.51
6–10	1.64	11.11	6.77
11–20	11.48	10.41	10.91
21–30	12.30	9.72	10.9
31–40	5.74	4.17	4.89
41–50	38.52	29.86	33.84
51–60	4.10	4.17	4.14
61–70	2.46	0.69	1.50
71–80	6.56	8.33	7.52
81–90	0.00	0.00	0.00
91–100	7.38	21.53	15.04
Total	100.00	100.00	100.00

### Correlations between TPB variables and patterns in farmers’ opinions

**Fig 2:**
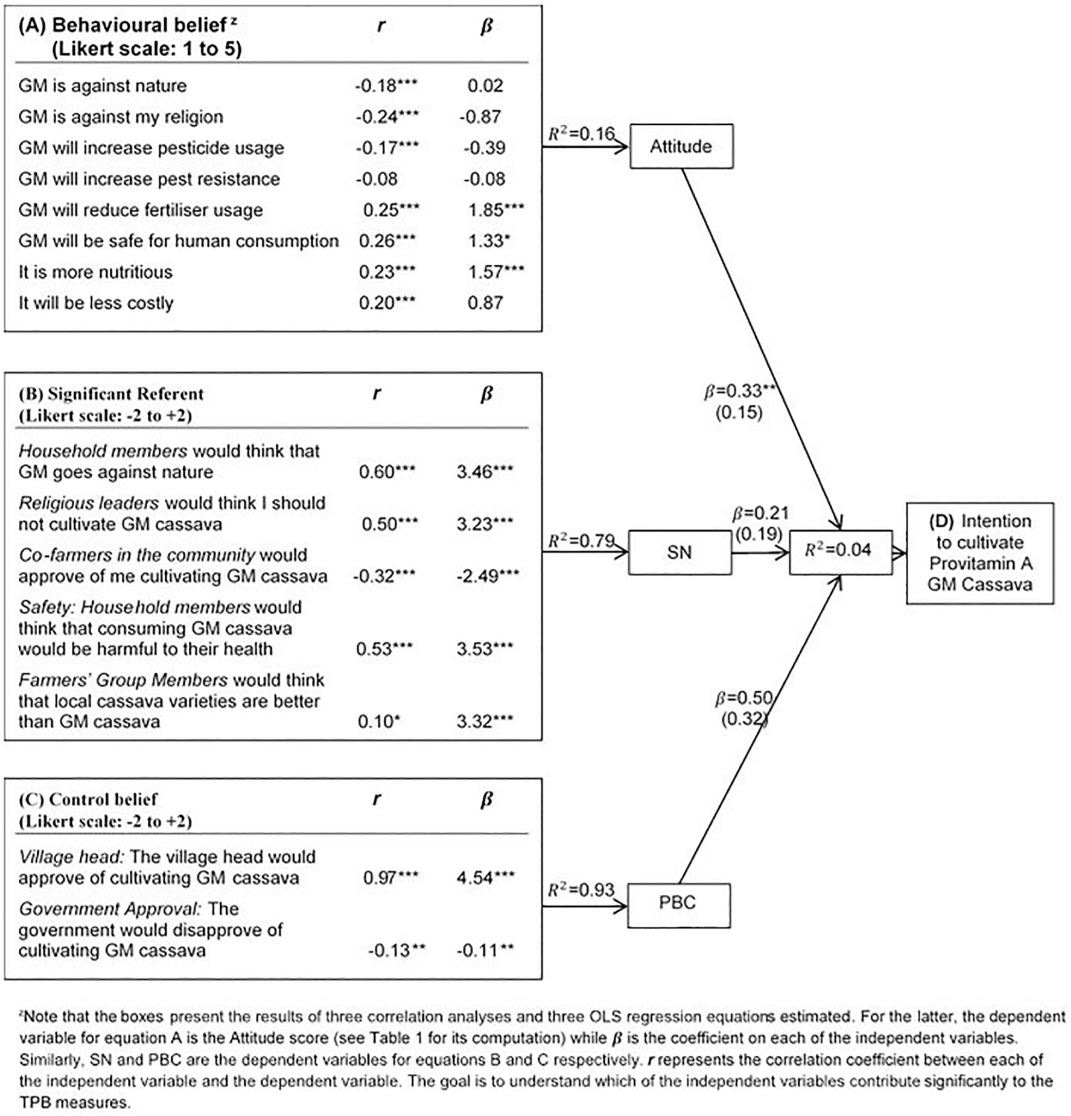
TPB conceptual path to farmers’ intentions towards cultivating provitamin A GM cassava (***p<0.*01*, **p<0.*05*, *p<0.*10)*.

All questions used to examine TPB variables were measured on 5-point Likert scales and the scores are computed as proposed by the TPB discussed in the previous Section. These questions were reversed to the same direction before computing each score so as to ensure consistency in interpretation such that the mid-point (zero) represents the point of neutrality. For *SN* and *PBC*, each outcome evaluation statement is worded following belief statements shown in Fig 2. Eight opinion statements were utilised in constructing attitude, five for *SN* and two for *PBC*. Thus the possible range of total score for attitude (∑*b*_*i*_*e*_*i*_) is [(5*±2)*8 = -80 to +80], for *SN* (∑*n*_*i*_*m*_*i*_) is [(5*±2)*5 = -50 to +50] and for *PBC* (∑*c*_*i*_*p*_*i*_) is [(5*±2)*2 = -20 to +20]. Computed scores range from -22 to +51 for attitude, -45 to +38 for *SN* and -10 to +10 for *PBC*.

Mean attitude score for all farmers surveyed is 23 (±12) which indicates a positive and supporting attitude towards cultivating Provitamin A GM cassava. However, this is significantly different across the two states at 1% level, where farmers in Oyo (+26) have more positive attitude on average than those in Benue (+20). Positive attitude and high intention found in these two states is consistent with other studies that have generally shown that while Europeans remain negative about GM, the public in the US and developing countries tend to be positive about the technology [61–64]. Meanwhile, one key question is whether or not this positive attitude has a significant influence on farmers’ intentions.

To investigate this area, we first carry out correlation analyses to examine which behavioural antecedent has a bearing on other TPB variables. The level of correlation is shown by Pearson correlation coefficient (*r*) while *β* represents association between each behavioural antecedent and other TPB variables (Table 4). Secondly, we examine the association between intention and other TPB variables. Fig 2 shows the TPB conceptual path to farmers’ intentions toward cultivating Provitamin A GM cassava where results of four ordinary least square regressions (OLS) are diagrammatised. In model A, various behavioural beliefs are included as covariates and the dependent variable is attitude. Similarly, model B tests the influence of normative beliefs about opinions of significant referents on an individual’s perception of social pressure (*SN*). Model C also examines the association between control beliefs and *PBC* while model D investigates the influence of attitude, *SN* and *PBC* on farmers’ intentions to cultivate Provitamin A GM cassava. Along the path, *R*^*2*^ is reported as a measure of goodness of fit for each model.

**Table 4:** Correlation between TPB variables (Pearson’s *r*).

	**Attitude**	**Subjective Norm**	**PBC**
Subjective Norm	0.1653***		
PBC	0.1514***	-0.2137***	
Intention	0.1666***	0.0757	0.1012

***1% significance level.

Contrary to the expectation based on the behavioural theory applied, TPB variables are weakly correlated with farmers’ intentions to cultivate Provitamin A GM cassava. The correlation coefficients show that attitude and PBC are positively correlated and both are also positively correlated with intention. This shows that the more control farmers feel towards being able to make independent decisions on cultivating Provitamin A GM cassava, the more positive attitude they have and hence the more they are willing to cultivate the crop. However, the positive sign on the correlation coefficients between SN and attitude as well as SN and intention proves contrary to *a priori* expectation since we expect that social pressure is likely to be negatively associated with farmers’ attitude and intention to cultivate Provitamin A GM cassava.

Further, the regression outputs presented in Fig 2 show various belief components that significantly contributed to farmers’ attitude, *SN* and *PBC*. Among the behavioural beliefs (model A), the belief that GM is against nature and religion is negatively correlated with farmers’ attitude as expected since ethical concerns can minimise optimism about GM [65]. [66, 67] also noted that the public can become ambivalent about GM by tagging the technology with connotations such as ‘realms that should be left to God and God alone’, ‘playing God’ or ‘trying to displace the first Creator’. While it would be expected that producers will be interested in profitability, the positive coefficient on the behavioural belief regarding price factor (model A) indicates that farmers still have a positive attitude even if Provitamin A GM cassava is cheap and affordable to consumers. This results is consistent since farmers in rural areas consume what they produce, thus are jointly producers and consumers at the same time.

Similarly, coefficients on perception of environmental risk and input requirement (pesticide and fertiliser usage) have signs as expected. The results show that although farmers’ perception of the risk of increased pest resistance and pesticide usage is negatively associated with attitude, it does not significantly contribute to their attitudes [68] also found that pesticide expenditure did not contribute significantly to the adoption rate subsequent to the introduction of Bt Eggplant in India. However, if less fertiliser application is required for GM cassava compared with conventional varieties, farmers’ attitude is more likely to be positive towards cultivating Provitamin A GM cassava. The perception of the nutrition benefit of Provitamin A GM cassava has a relatively large coefficient which is significant and positively associated with farmers’ attitude. It indicates that the perception of the nutritional benefits is positively correlated with farmers’ attitudes toward cultivating Provitamin A GM cassava. These results are similar to those of [20] where a higher benefit perception was shown to increase the likelihood of purchasing GM banana among farm households in Uganda.

In the case of *SN* (model B), concerns about the opinion of other household members, religious leaders and farmers’ group members significantly contributed positively to the overall perception of social pressure while opinion of co-farmers within a community minimises the perception of social pressure towards cultivating Provitamin A GM cassava. Consistently with the TPB, these normative beliefs strongly predicted about 79% of variance in *SN* while control beliefs also strongly predicted about 93% of variance in the overall perception of level of control (*PBC*) farmers’ feel they have toward cultivating Provitamin A GM cassava if and when made available (model C). On the other hand, behavioural belief components do not predict farmers’ attitude very well since only 16% of variance in attitude was predicted (model A). Even though the result presented in Table 4 shows that attitude is significantly and positively correlated with farmers’ intentions, this is different at a state level when socio-economic variables are controlled for.

Table 5 presents OLS regression results examining the association between TPB variables and intention at a state level. The basic model where the pool data were utilised (3) shows that attitude, *SN* and *PBC* weakly predicted farmers’ intention where only 4% of variance in intention was predicted by the TPB variables (3). With the inclusion of other covariates, the *R*^*2*^ increased significantly for Benue (4) and Oyo (5) models. Results from these more robust models are similar to those of the basic models (1 and 2). Consistently with the theory applied, the result shows that attitude is positively and significantly associated with farmers’ intention in Benue. In contrast, *SN* and *PBC* are the significant determinants of intention in Oyo (5).

**Table 5:** Models of farmers’ intention towards cultivating provitamin A GM cassava (OLS regressions).

Variable	Dependent Variable: Intention					
	Basic (Benue)	Basic (Oyo)	Basic (POOL)	Benue With socio-economic variables	Oyo With socio-economic variables	POOL With socio-economic variables
	1	2	3	4	5	6
	Coeff. (std err)	Coeff. (std err)	Coeff. (std err)	Coeff. (std err)	Coeff. (std err)	Coeff. (std err)
Attitude	0.34 (0.16)**	0.14 (0.31)	0.33 (0.15)**	0.31 (0.18)*	0.22 (0.33)	0.18 (0.17)
*SN*	0.13 (0.21)	0.68 (0.35)*	0.21 (0.19)	0.05 (0.24)	0.80 (0.36)**	0.27 (0.21)
*PBC*	0.09 (0.51)	1.10 (0.47)**	0.50 (0.32)	-0.23 (0.58)	1.19 (0.48)**	0.65 (0.35)*
State (Benue = 1, otherwise 0)	-	-	-	-	-	-5.97 (5.03)
Gender (Male = 1, otherwise 0)	**-**	**-**	**-**	9.66 (5.72)*	2.77 (6.08)	3.40 (4.27)
Married (married = 1, otherwise 0)	**-**	**-**	**-**	6.91 (7.10)	-11.77 (8.67)	-2.91 (5.76)
Age (years)	**-**	**-**	**-**	-0.48 (0.18)***	-0.08 (0.19)	-0.25 (0.13)*
Education (years)	**-**	**-**	**-**	-1.64 (0.55)***	0.73 (0.62)	-0.47 (0.43)
Household size	**-**	**-**	**-**	0.63 (0.51)	-0.25 (0.63)	0.07 (0.42)
Wealth Index	**-**	**-**	**-**	-0.67 (2.10)	0.22 (2.44)	0.06 (1.67)
Cassava land area (ha)				0.15 (0.42)	-3.83 (1.19)***	-0.42 (0.45)
Heard about GM before survey	**-**	**-**	**-**	-5.72 (15.24)	18.29 (8.15)**	9.53 (6.87)
Trust in government extension officers (1: Distrust very much = 1 to 5: Trust very much)	**-**	**-**	**-**	1.91 (4.15)	-2.51 (3.05)	-1.62 (2.49)
Constant	35.73 (4.56)***	40.87 (8.89)***	37.12 (4.09)***	50.71 (22.58)**	65.16 (20.38)***	64.46 (14.38)***
*N*	121	143	264	101	139	240
*R*^2^	0.05	0.04	0.04	0.19	0.17	0.08

***1% significance level

** 5% significance level

*10% significance level

(): Robust standard error

The positive coefficient obtained on *SN* in Oyo is contrary to expectation while the positive coefficient on *PBC* (5) suggests that the more farmers feel that they have control to actualise their intentions, the less likely they think that government will disapprove of cultivating GM cassava, which is plausible. Also in the pool model with socio-economic variables (6), only *PBC* significantly influenced intention. Trust in government may be a driving factor since an opinion statement in this regard (1: distrust very much to 5: trust very much) shows that farmers in both states trusted government agricultural extension officers very much (mean: 4.57±0.75) on average.

Meanwhile, the negative sign obtained on the coefficient (albeit not significant) for the trust variable is ambiguous (5 and 6). On one hand, this result is contrary to the evidence emerging from other studies. On the other, it may reveal that the aspect of trust being measured matters since trust is a broad concept that may be difficult to capture in a snapshot. While [15] found that trust in regulatory authorities has a significant effect on consumer demand for Provitamin A GM cassava in Brazil we found no significant effect of trust in government agricultural extension officers on farmers’ intentions to cultivate Provitamin A GM cassava in Nigeria. Beside this, the regression results also indicate that less educated younger male farmers are more likely to have higher intentions to cultivate Provitamin A Provitamin A GM cassava in Benue (4). In Oyo, those farmers who have heard about GM *ex ante* before the survey and those who cultivated smaller cassava land area are more likely to have higher intentions to cultivate Provitamin A GM cassava (5). It is not surprising that prior awareness about GM is higher in Oyo state and that this variable also has a positive association with intention. This may be because of a higher chance of farmers getting access to agricultural experts since several agricultural research institutes such as the International Institute for Tropical Agriculture (IITA) are located within the state.

While results from both correlation and regression analyses indicate that TPB variables are weakly associated with farmers’ intentions toward Provitamin A GM cassava, it could also reflect that there are some other factors missing in our study that may be important to farmers in developing their opinions toward cultivating the crop. Such factors could include other agronomic (e.g. yield and early maturity) and processing traits (e.g. dry matter content, starch content, pounding ability and fiber content) which were omitted. Also, limited time interval between information transfer and interview may still leave an individual with uncertainties since there could be a dynamic process of rethinking and learning. Our results show only immediate reactions of farmers to the concept of GM and Provitamin A GM cassava. Therefore, the results are interpreted within the context of these caveats.

### Clusters of farmers based on opinions about provitamin A GM cassava

Well-defined groups exist when the number of clusters is three, which is the point at which CH-statistic (Eq 2) is optimal (140.23). We categorized the sample into three distinct clusters using the TPB variables that reflect different levels of opposition towards GM and Provitamin A GM cassava. Mean scores for attitude, *SN* and *PBC* for the three clusters are presented in Table 6. The mean attitude score for the first cluster is the highest (29.58) and above the neutral value of zero. Also, this cluster of farmers has a very low concern about opinions of significant others (mean *SN*: -0.61) and the strongest perception of control over ability to actualise their intentions (mean *PBC*: 8.83). This indicates that farmers in the first cluster are most positive about GM and are less likely to face opposition from their family members, religious leaders or members of farmers’ groups, thus the cluster is referred to as ‘low opposition’ cluster. Almost half of the farmers surveyed are in this cluster.

**Table 6:** TPB variables and socioeconomic characteristics by cluster.

	**Cluster 1**	**Cluster 2**	**Cluster 3**	t-test*
	Early Adopters	Late Majority	Laggards	
	n = 132	n = 84	n = 71	
	46%	29%	25%	
	*Low Opposition*	*Medium Opposition*	*High Opposition*	
	High Attitude, Low SN, High PBC	High Attitude, High SN, Low PBC	Low Attitude, Low SN, High PBC	
Attitude	29.58 (6.40)	25.49 (7.33)	5.91 (9.57)	Aa, Ba, Ca
SN	-0.61 (6.05)	12.66 (7.39)	-0.47 (9.94)	Aa, Ca
PBC	8.83 (2.42)	2.99 (7.31)	6.13 (5.14)	Aa, Ba, Ca
Intention to cultivate Provitamin A GM cassava (% cassava land area)	50.45 (30.07)	52.13 (28.87)	41.30 (22.81)	Bb, Cb
State (Benue = 1, Oyo = 0)	0.37	0.49	0.73	Ac, Ba, Ca
Gender (Male = 1, Female = 0)	0.71	0.60	0.63	Ac
Married	0.86	0.89	0.87	
Age (years)	48.75 (15.43)	49.71 (15.62)	45.20 (14.39)	Cc
Education (years)	6.12 (5.36)	6.01 (5.65)	8.26 (5.44)	Ba, Cb
Household size	7.88 (4.20)	8.35 (4.79)	10.27 (5.46)	Ba, Cb
Wealth Index	1.50 (1.35)	1.57 (1.44)	2.16 (1.37)	Ba, Cb
Cassava land area(ha)	1.98 (2.72)	2.53 (8.28)	3.12 (7.14)	
Heard about GM *ex ante* (1, otherwise = 0)	0.08	0.04	0.08	
Trust in government extension officers (1: Distrust very much = 1 to 5: Trust very much)	4.48 (0.74)	4.63 (0.77)	4.68 (0.75)	Bc

*a– 1% significance level, b– 5% significance level, c– 10% significance level, () standard deviation, A–One-sided t-test between clusters 1 and 2, B–One-sided t-test between clusters 1 and 3, C—One-sided t-test between clusters 2 and 3

**Fig 3:**
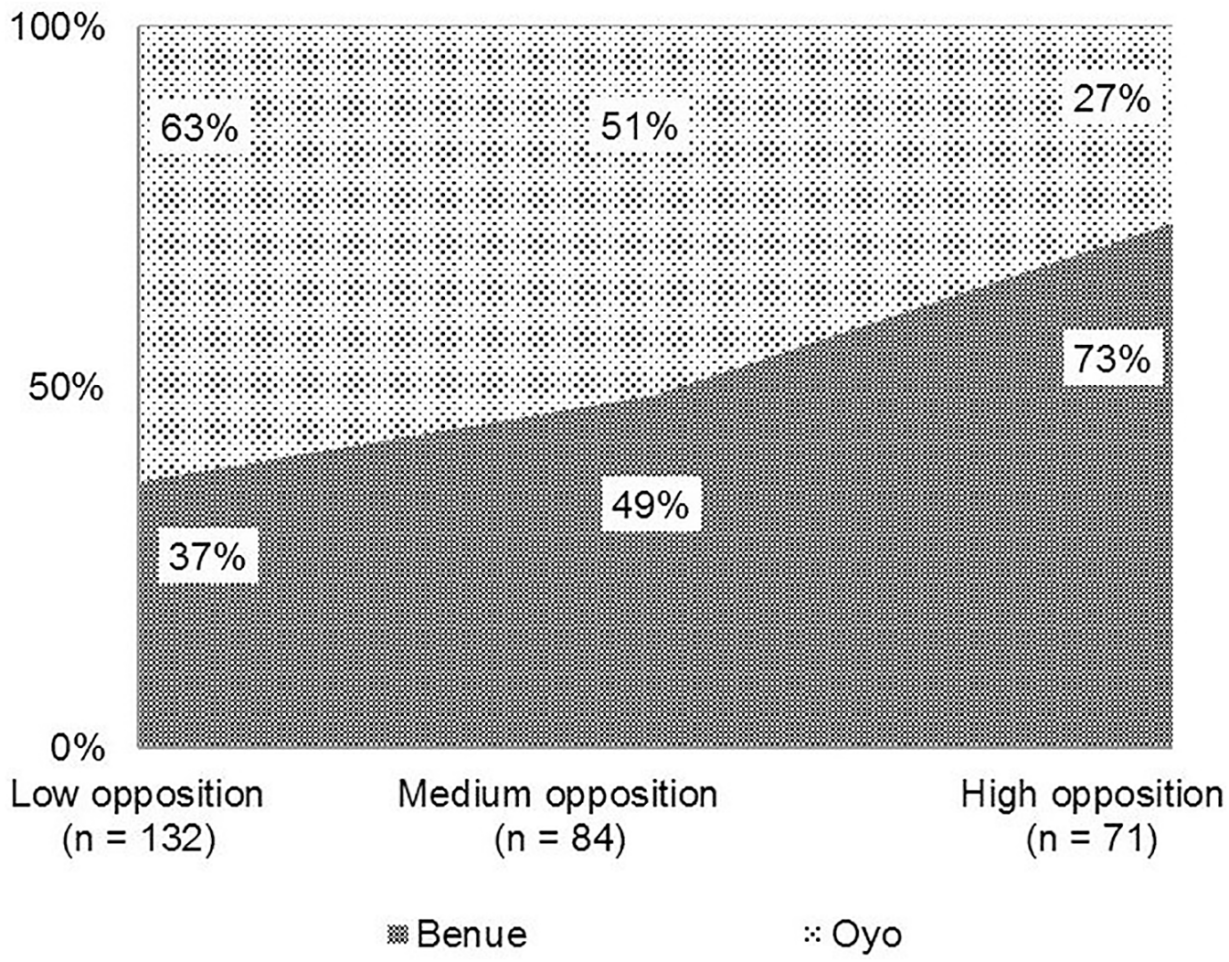
Percentage of farmers by cluster and state.

Opinions of this cluster of farmers have some relevance in the Rogers’ theory of diffusion of innovations. [69] suggested that early adopters are more likely to be opinion leaders whose adoption decision is likely to decrease uncertainties about a technology. However, in the case of Provitamin A GM cassava, this low opposition cluster is characterised by poorer farmers with lower wealth index compared with those of other clusters. Even though these farmers may not be opinion leaders within their communities they are the most positive about Provitamin A GM cassava. Therefore, they may be more likely to adopt the crop first when introduced. On average, farmers in ‘low opposition’ cluster have about 6 years of formal education and are about 49 years old where 8% of them have heard about GM *ex ante*, 71% are male and 86% are married. A majority of farmers in this cluster are in Oyo state (63%) (Fig 3).

Farmers in the second cluster (29% of farmers surveyed) also have an average attitude score above the neutral range (25.49) but they are the most concerned about significant others’ opinions (*SN*: 12.66) and they feel a very low sense of control to actualise their intentions (mean *PBC*: 2.99). Compared with other clusters, this group of farmers is particularly concerned about the opinion of religious leaders on GM. For the statement ‘*religious leaders will think I should not participate in cultivating Provitamin A GM cassava’*, cluster 2 farmers revealed the strongest supporting response (mean: 1.62). This indicates that although members of this cluster themselves are more positive about Provitamin A GM cassava, they are more likely to face oppositions from significant referents compared with farmers in the first cluster. As a result, the second cluster is referred to as the ‘medium opposition’ cluster.

On average, farmers in this cluster are about 50 years old with about 6 years of formal education where 89% are married, 60% are male, and 4% have heard about GM *ex ante*. About half of farmers in the medium opposition cluster are in Benue state and the other half in Oyo state. [69] noted that the ‘late majority’ adopter class consists of members of a social system who typically wait until most of their peers adopt an innovation. The late majority adopters are usually susceptible to adopting an innovation due to peer pressure [70]. This is similar to the medium opposition cluster where farmers have a higher *SN* on average relative to other clusters.

The third cluster (25% of farmers surveyed) represents a group of farmers that are more likely to have a ‘high opposition’ to Provitamin A GM cassava because despite the fact that they have the least concern about opinions of significant referents and feel a positive sense of control towards actualising their intentions, they still have the least positive attitude on average (5.91) relative to other clusters. In addition, farmers in the ‘high opposition’ cluster are wealthier and are the most educated with about 8 years of formal education on average. Majority of them (73%) are located in Benue state. While on average, the low opposition cluster cultivated about 2ha of land with cassava, high opposition cluster cultivated about 3ha of land with cassava. This cluster of farmers is likely to end up as a group of ‘laggards’ who have traditional views and would become more skeptical about GM technology; thus, may wait for the technology to work before adopting it.

Using socio-economic variables described above, we constructed a model to predict cluster membership. A multinomial logit model was estimated and the results are presented in Table 7. Attitude significantly influenced the group membership where the result indicates that low opposition farmers are more likely to have a positive attitude while high opposition farmers are more likely to have a negative attitude. Larger household size increases the likelihood of being a high opposition farmer which is surprising since this group is the least concerned about the opinion of significant others. Further, mean values for normative belief statements are significantly different across clusters. Medium opposition farmers are strongly concerned that (i) household members will think GM is against nature (1.55), (ii) religious leaders will think a farmer should not participate in cultivating GM cassava (1.62) and (iii) household members would think consuming GM cassava is not safe for consumption (1.42). High opposition farmers are the least concerned about the opinion of religious leaders (0.76) and household members (0.17) while low opposition farmers are the least concerned about the opinion of farmers’ group members (-0.89). Low opposition farmers strongly believe that the village head and co-farmers in the community will approve of cultivating Provitamin A GM cassava while medium opposition farmers disagreed on average that co-farmers will approve of GM.

**Table 7:** Parameter estimates from a multinomial logit model estimation for group membership (Base outcome: Cluster 2).

	Cluster 1	Cluster 3
	*Low Opposition*	*High Opposition*
Variable	Coeff. (std err)	Coeff. (std err)
Attitude	0.09*** (0.03)	-1.20*** (0.32)
*Locational attribute*		
State (Benue = 1, Oyo = 0)	-0.59 (0.43)	-0.01 (1.25)
*Socio-economic characteristics*		
Gender (male = 1, female = 0)	0.54 (0.36)	1.89 (1.42)
Married (1, otherwise = 0)	-0.46 (0.55)	-1.50 (1.65)
Age (years)	-0.01 (0.01)	-0.04 (0.04)
Education (years)	0.30 x 10^−3^ (0.04)	-0.15 (0.15)
Household size	-0.02 (0.04)	0.19* (0.10)
Wealth Index	0.11 (0.15)	-0.45 (0.56)
Cassava land area (ha)	-0.02 (0.04)	-0.01 (0.06)
Heard about GM *ex ante* (1, otherwise = 0)	1.01 (0.72)	0.90 (3.80)
Trust in government extension officers	-0.33 (0.24)	-0.48 (0.87)
Constant	-0.10 (1.47)	23.01*** (7.09)
*N*	*260*	
*Pseudo R*^*2*^	*0*.*5184*	
*Log-likelihood*	*-132*.*5923*	

***1% significance level

*10% significance level, () standard error

## Conclusions and implications

This paper has investigated farmers’ attitude and intention toward cultivating Provitamin A GM cassava in rural Nigeria. A survey of farmers’ opinions about the technology was conducted with a random sample of 288 cassava farmers in two major cassava producing states in Nigeria. Opinions obtained reflect farmers’ immediate reactions after receiving information about the concept of GM. An application of the theory of planned behaviour (TPB) shows that smallholder farmers in rural Nigeria are highly intending to cultivate Provitamin A GM cassava. There is generally a positive attitude towards the technology. Perceptions about the nutritional benefits and low fertiliser requirement significantly contributed to farmers’ positive attitude towards cultivating Provitamin A GM cassava while perceptions of the environmental risk of increased pesticide usage minimises this attitude. Therefore, high input requirements represent a major threat to the adoption of Provitamin A GM cassava among rural farmers in Nigeria. While we recognise that these immediate behavioural reactions may change with learning and availability of more information, it will be interesting for further research to explore the dynamics.

A *k-*means cluster analysis of the TPB variables in terms of farmers’ opinions toward cultivating Provitamin A GM cassava was conducted leading to the identification of three distinct clusters of farmers. The first cluster consists of the *low opposition* farmers who have significantly high and supporting attitude towards cultivating Provitamin A GM cassava and they are the least concerned about the opinion of significant referents. Most of the low opposition farmers are located in Oyo state in the South-west; they are poorer and planted the smallest area of land with cassava. This group of farmers who perceived low social pressure from significant others is likely to constitute early adopters when Provitamin A GM cassava is introduced. As suggested elsewhere poverty with regards to a lack of high quality foods at home could be responsible for positive attitude such as the high willingness to purchase and try GM foods found among Columbian consumers [71].

*Medium opposition* farmers are located in both Oyo and Benue states equally. Although these farmers have high and positive attitude towards cultivating Provitamin A GM cassava but the high subjective norm suggests that they are the most concerned about the opinion of significant referents on GM cassava. Medium opposition farmers are particularly concerned that religious leaders in the community will think that a farmer should not participate in cultivating GM cassava and that their household members will also think GM is against nature and that it is not safe for consumption. This is of policy importance because ethical and safety concerns represent a significant barrier to the adoption of Provitamin A GM cassava. Medium opposition farmers are likely to be the ‘late majority’ adopters who will wait to observe the trend and adopt or not adopt the GM technology due to peer pressure. Thus, timely and appropriate communication strategy is important to suppress misinformation about GM among this group of farmers. Since religious leaders’ opinion matters most to this segment of cassava farmer population, inclusive programmes to improve knowledge about GM among Christian and Islamic leaders may prove to be an effective communication strategy to minimise oppositions due to misinformation.

*High opposition* farmers are wealthier and more educated than farmers in other clusters. Almost three quarter of this group of farmers are located in Benue state. They strongly feel that they have control over their decisions to adopt Provitamin A GM cassava and are not highly concerned about the opinion of significant others but they just generally have low attitude towards cultivating the crop. These farmers are likely to be the laggards who would have traditional views which may be hard to change.

Low and medium opposition farmers constitute a majority of the cassava farmer population in the two states and they may be more likely to adopt Provitamin A GM cassava varieties. Therefore, educational-based strategies such as training programmes are important in providing good and balanced sensitisation among this segment of the population. Also, the high opposition group is more likely to be influential in rural communities and thus can influence opinions of low and medium opposition farmers. As such, it is necessary for policy to target intensive training programmes at the high opposition farmers in order to provide them with balanced views about GM before they get mixed information.

Results of the correlation analysis among TPB variables does not support the *a priori* assumption that attitude, subjective norm and perceived behavioural control will strongly predict intention. In interpreting these results, it is important to note two key caveats. First, knowledge about GM among rural farmers surveyed is limited and measures of TPB variables reported here reflect mainly farmers’ immediate reactions to the concept of GM. Again, although the study shows that very few farmers were informed about GM *ex ante*, the source and type of information they heard on GM should have been controlled for since these may influence their perceptions. Second, the result might have been influenced by the problem of omitted variables where agronomic traits such as yield and early maturity as well as processing attributes such as dry matter content, starch content, pounding ability and fiber content were not listed in the survey. This may be one reason why even though attitude is positively associated with intention; it weakly predicted the variance in farmers’ intentions. An application of a stated preference technique considering farmer’s trade-offs among various production, consumption and processing attributes of Provitamin A GM cassava can remove this limitation.

## Appendix (further comments)

1. This is for research purpose only and has nothing to do with HarvestPlus intervention programmes. Officially released HarvestPlus Provitamin A cassava varieties in Nigeria have been bred through conventional methods and not GM method. Therefore, please note that the GM cassava referred to in this article is not HarvestPlus or IITA’s Provitamin A cassava in Nigeria. More information about HarvestPlus program can be found on www.harvestplus.org. This paper should not be the basis for misinformation or miscommunication about HarvestPlus program in Nigeria or elsewhere. All opinions and errors are authors’ own.

## Supporting information

S1 Box Information communicated to farmers on GM and provitamin A GM cassava. (PDF)
