# Supplementary material for: Will farmers intend to cultivate Provitamin A genetically modified (GM) cassava in Nigeria? Evidence from a k-means segmentation analysis of beliefs and attitudes
Source: PLoS One. 2017 Jul 11;12(7):e0179427. doi: 10.1371/journal.pone.0179427 (PMC5507399; doi:10.1371/journal.pone.0179427)
Supplement: S1 Box — (PDF) [file pone.0179427.s001.pdf]

S1. Box: Supporting Document

Box 1: Information communicated to farmers on GM and Provitamin A GM Cassava

What is Conventionally Bred Cassava?

Cross-breeding is not the same thing as Genetic Engineering or GMO!

Cross-breeding: To produce [an organism] by the mating of individuals of different breeds, varieties or species but of the **same kind**.

The acronym GMO stands for 'Genetically Modified Organism'. GMO is the offspring or baby or hybrid that is created out of mixing genes from organisms of **different kinds**

Conventionally bred cassava is a cassava variety developed through conventional breeding. This can be termed "cassava-to-cassava".

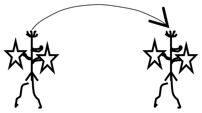

Conventional breeding involves combining two different cassava varieties: for example, scientists can combine one cassava variety A (which is high in vitamins but low yielding) with another cassava variety B (which is high yielding but low in vitamins) to get a new cassava variety C (which is both high yielding and high in vitamins).

For explanation

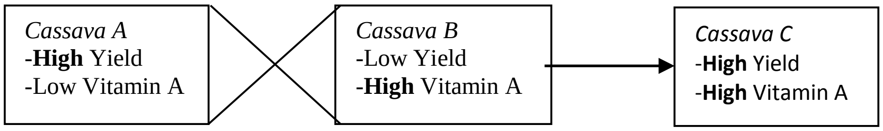

Transgenic/GM refers to a plant or animal that contains genes not normally found in that particular plant or animal.

Transgenic/GM cassava is a cassava variety developed through 'transgenic breeding'.

Provitamin A Transgenic Cassava breeding involves for example combining a cassava variety X (which is high yielding but low in vitamins) with *[some species e.g. plant other than cassava]* (which is high in vitamins) to get a new cassava which is high yielding and high in vitamins. This can be termed "another plant-to-cassava".

For explanation *Provitamin A GM Cassava*

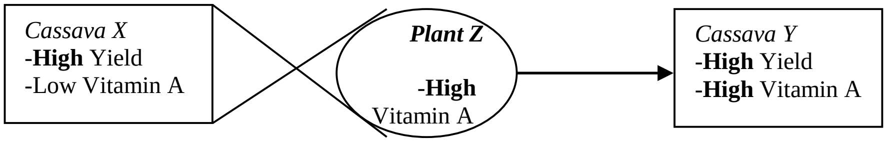

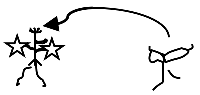

Vitamin A is important for the health of everyone in the family, and especially children. It reduces the risk of some diseases and improves eyesight. Other foods apart from Provitamin A GM Cassava that have a lot of Vitamin A include: meat, milk, eggs, those that are very orange, such as paw-paws, carrots, pumpkins, and those that are very green, such as spinach, bondwe, and pumpkin leaves. Also, red palm oil contains vitamin A.
